# Contrasting Responses of Oceanic and Coastal *Synechococcus* to Iron Limitation and Warming Interactions

**DOI:** 10.1111/1758-2229.70158

**Published:** 2025-07-18

**Authors:** Ran Duan, Min Xu, Xiaopeng Bian, Conner Y. Kojima, Shengwei Hou, Qiang Zheng, Seth G. John, David A. Hutchins, Fei‐Xue Fu

**Affiliations:** ^1^ Department of Biological Sciences University of Southern California Los Angeles California USA; ^2^ School of Marine Science and Engineering Hainan University Haikou China; ^3^ Department of Earth Sciences University of Southern California Los Angeles California USA; ^4^ Department of Ocean Science and Engineering Southern University of Science and Technology Shenzhen China; ^5^ College of Ocean and Earth Sciences Xiamen University Xiamen China

**Keywords:** Fe limitation, ocean warming, picocyanobacteria, *Synechococcus*, transcriptome

## Abstract

This study explored the contrasting physiological and transcriptional responses to iron (Fe) and warming temperature interactions in two South China Sea *Synechococcus* isolates belonging to clade II from the open ocean and CB5 from the coastal ocean. The two picocyanobacterial strains utilised contrasting photosynthesis, Fe uptake, and nutrient acquisition strategies to cope with Fe limitation. In the oceanic strain, moderate warming under Fe limitation upregulated expression of photosynthesis and nutrient and Fe transport genes, increasing its growth and photosynthesis. In contrast, gene expression under low Fe in the coastal strain was less affected by warming. The oceanic isolate exhibited substrate regulation of Fe acquisition and preferred organic nutrient sources. The coastal strain had a much higher Fe quota, faster turnover of the D1 gene in photosystem II, and was optimised for inorganic nitrogen sources. Both strains showed multi‐tiered Fe uptake strategies and general stress responses to heat shock and oxidative stress. In general, gene regulation in the oceanic strain responded more effectively to both stressors than in the coastal isolate. Fe‐temperature interactions in both strains are complex and may lead to synergistic and antagonistic responses, potentially influencing global biogeochemical cycles in warmer oceans.

## Graphical abstract

Oceanic *Synechococcus* respond to the interactive effects of iron (Fe) limitation and ocean warming by regulating photosynthesis, nutrient metabolism, and heat shock gene expression, while a coastal strain has fewer regulatory mechanisms. The oceanic strain may be a superior competitor with more comprehensive and flexible responses in Fe‐poor subtropical open ocean regimes. Fe‐temperature interactions may lead to complex synergistic and antagonistic responses, with significant implications for future plankton community dynamics and marine biogeochemistry.

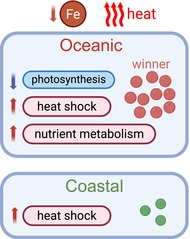

## 1 Introduction

The Sixth Assessment Report (AR6) of the Intergovernmental Panel on Climate Change (IPCC) has stated that global temperatures have risen by ~1.5°C due to increased CO_2_ emissions since the Industrial Revolution. In the ocean, warming can intensify stratification, especially in subtropical and tropical regions. This hinders the exchange of nutrients between surface and deep waters, reducing supplies to primary producers (Hutchins and Fu 2017). This includes vertical advective fluxes of iron (Fe), the most important trace element limiting primary production in the ocean (Tagliabue et al. 2017). At the cellular level, Fe plays a role in many crucial metabolic pathways, including photosynthesis, carbon fixation, and nitrogen assimilation (Kranzler et al. 2013). Warmer temperatures and Fe availability can either individually or interactively affect biological enzymatic activity and central metabolism (Hutchins and Boyd 2016).

The marine *Synechococcus* genus is one of the most dominant picocyanobacterial groups, as it is widely distributed across various ocean regions and contributes around 17% of marine net primary production (Lee et al. 2019). Previous studies have investigated the evolutionary adaptations of *Synechococcus* clades and ecotypes to environmental factors (Lee et al. 2019; Ahlgren et al. 2020). The key drivers of niche specialisation in this genus are Fe, temperature, light, and nutrients (Lee et al. 2019; Sohm et al. 2016; Kling et al. 2023).

Open ocean and coastal areas typically have different environmental conditions, including variations in Fe and temperature. The main source of Fe in oceanic areas is dust from the atmosphere, and surface concentrations are very low, usually less than 0.2 nM (Johnson et al. 1997). Coastal areas receive Fe from multiple sources, such as river input and upwelling. Thus ambient Fe concentrations are higher than in the open ocean, although phytoplankton iron limitation can still occur in some nearshore regions (Capone and Hutchins 2013). The temperature in oceanic areas is also more stable compared to coastal waters because the open ocean has a greater heat capacity, which helps moderate temperature changes, while coastal waters are more affected by seasonal temperature fluctuations, wind patterns, and ocean currents (Wang et al. 2020). Specifically, the warming rate in the South China Sea is about twice the global ocean mean rate (Fang et al. 2006).

Previous studies have explored differences in growth, photophysiology, and proteomic allocation for photosynthesis and Fe acquisition in *Synechococcus* isolated from oceanic and coastal areas of the North Atlantic, which have varying concentrations of key nutrients like nitrogen (N) and phosphorus (P) (Mackey et al. 2015). Considering the complexity of natural environments, we performed a multi‐stressor experiment in the laboratory using *Synechococcus* isolates from adjacent coastal and oceanic areas of the South China Sea to explore how different ecotypes respond to environmental changes; proteomic responses in this experiment were presented in Schiksnis et al. (2024). Here, we investigated differences in transcriptional networks in both nearshore and oceanic *Synechococcus* clades in response to iron and warming interactions in these contrasting ocean regimes.

**FIGURE 1:**
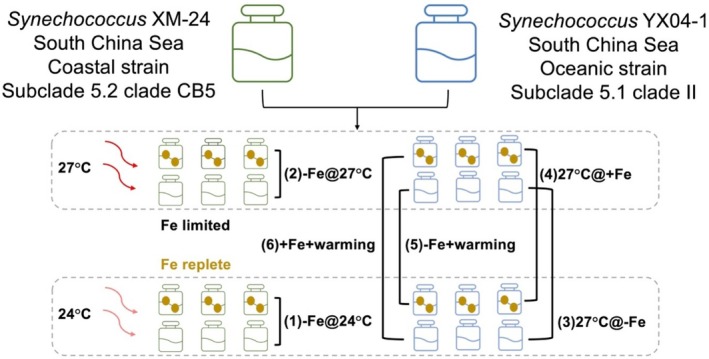
Experimental design schematic for both *Synechococcus* strains utilised in the study. Schematic overview of the coastal strain (*Synechococcus* XM‐24, green) and the oceanic strain (*Synechococcus* YX04‐1, blue) from the South China Sea, each grown at two temperatures (24°C and 27°C) and under two Fe conditions (Fe‐limited or Fe‐replete). Six treatment comparisons were performed: (1) −Fe@24°C versus +Fe@24°C, (called “−Fe@24°C”), (2) −Fe@27°C versus +Fe@27°C (called “−Fe@27°C”), (3) −Fe@27°C versus −Fe@24°C (called “27°C@‐Fe”), (4) + Fe@27°C versus +Fe@24°C (called “27°C@+ Fe”), (5) −Fe@27°C versus +Fe@24°C (called “‐Fe + warming”), and (6) + Fe@27°C versus −Fe@24°C (called “+Fe + warming”). The gold circles represent Fe‐replete cultures, whereas flasks without circles represent Fe‐limited cultures. The dashed rectangles denote cultures in two temperatures (24°C and 27°C). The coastal strain, *Synechococcus* XM24, is denoted by green bottles, while the oceanic strain, *Synechococcus* YX04‐1, is represented by blue bottles. Taxonomic and isolation site information is included for the two strains. Further detailed description of experimental design can be found in the Experimental Procedure section and in Table S1.

The oceanic strain belongs to the widely distributed oligotrophic subcluster 5.1 clade II (YX04‐1), while the coastal strain belongs to subcluster 5.2 clade CB5 (XM24), which has been previously reported to be halotolerant in the coastal waters of the East China Sea and Chesapeake Bay (Choi and Noh 2009; Chen et al. 2006). We cultured these strains at two temperatures (24°C and 27°C, Figure 1, Table S1) that lie within the annual range of the present‐day South China Sea (Jin et al. 2023). At each temperature, we examined the effects of two Fe concentrations (2 and 250 nM) by measuring physiological responses such as growth, carbon fixation, and cellular Fe quotas. We also sequenced their transcriptomes across all four treatments to compare potential acclimation strategies in response to Fe‐warming interactions in these two strains. This study provides insights into how marine *Synechococcus* from taxonomically divergent coastal and oceanic clades regulate their transcriptional networks and short‐term responses to iron limitation in combination with moderate ocean warming in these adjacent but contrasting environments.

## 2 Results

### 2.1 Physiological Responses in Coastal and Oceanic Strains

**FIGURE 2:**
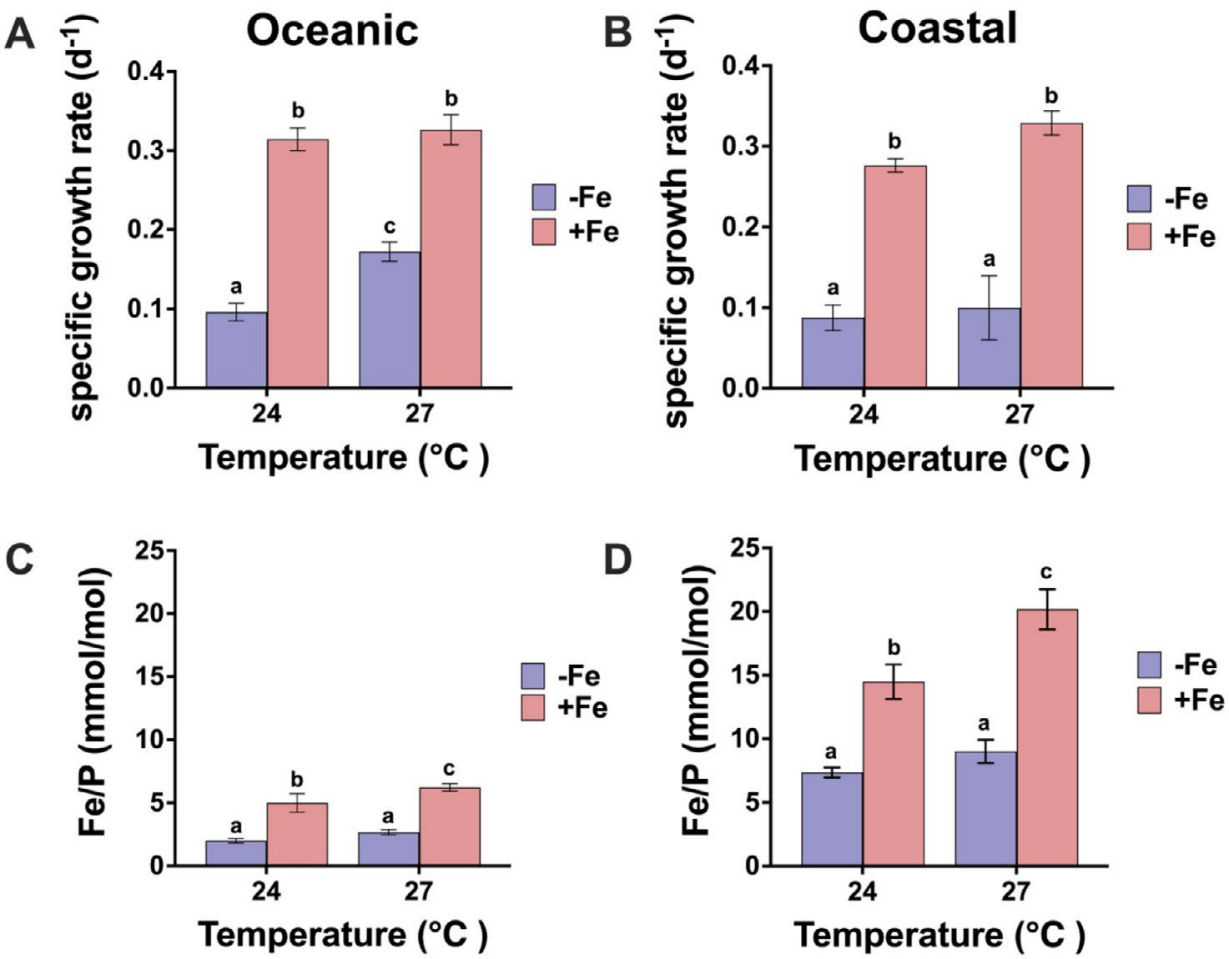
Specific growth rates and Fe quotas in the oceanic and coastal *Synechococcus* strains. Specific growth rates (d^−1^) for the oceanic strain (A) and the coastal strain (B) and Fe quotas as the iron (Fe) to phosphorus (P) ratio (mmol/mol) for the oceanic strain (C) and the coastal strain (D) in an experimental matrix of two temperatures (x axis) and two Fe conditions (pink replete and blue limiting). Error bars are standard deviations of triplicates. Mean values that do not share the same letter are significantly different from one another with *p* < 0.05 (tested by two‐way ANOVA).

In this section, we explored how Fe‐warming interactions affect physiology in both *Synechococcus* isolates, including growth, carbon fixation, and cellular Fe quotas. In both the coastal and oceanic *Synechococcus* isolates, Fe limitation led to a significant decrease (*p* < 0.05, tested by two‐way ANOVA and a Tukey multiple comparison test) in growth rates (Figure 2A,B), carbon fixation rates (Figure S1A,B), and Fe quotas (Fe/P, Figures 2C,D and Fe/C, S1C,D) at both high and low temperatures. Notably, the coastal strain had a much higher Fe quota than the oceanic strain, as the mean replete and limited Fe:P ratios of the coastal strain were about 3–4 fold higher than those of the oceanic strain (Table S2). In both the coastal and oceanic strains, the Fe quota (Fe/P) was increased with warmer temperatures under Fe replete conditions (Table S2, Figure 2).

Under Fe‐limited conditions, only the oceanic strain showed increased growth (~80%) with warming (Figure 2A, Table S2). Additionally, the growth of the oceanic strain was less impaired by Fe limitation at warming conditions. However, warming intensified the negative effects of Fe limitation on carbon fixation (Figure S1A). Carbon fixation rates showed minimal responses to warming (Figure S1A, 27°C@‐Fe). Under Fe limitation, carbon fixation rates were less affected by warming (Figure S1A) compared to the significant increase in growth. Warming alone, regardless of Fe availability, did not stimulate growth in the coastal strain.

### 2.2 Overview of Transcriptional Responses in Both Strains

**FIGURE 3:**
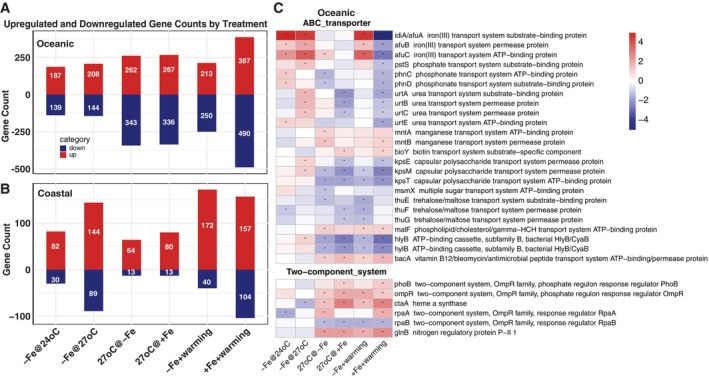
The differentially expressed gene (DEG) numbers in treatments for the oceanic strain (A) and the coastal strain (B). Red denotes upregulated DEGs, and blue denotes downregulated DEGs, and numbers of each are marked on bars. The heatmap of genes involved in the ABC transporter and two‐component system for the oceanic strain (C). The treatment columns include –Fe@24°C, –Fe@27°C, 27°C@‐Fe, 27°C@+ Fe, −Fe + warming, +Fe + warming. Detailed descriptions of each treatment comparison are given in the Methods. Each row denotes one gene with ID and annotation listed. The asterisk denotes differential expression with a fold change > 2 and an adjusted *p* < 0.05.

To investigate the overall profiles of transcriptomes in two *Synechococcus* isolates in response to Fe‐warming interactions, we identified the total differentially expressed genes (DEGs) and the most responsive DEGs under different conditions. The number of upregulated and downregulated genes that are differentially expressed in both strains is shown in Figure 3. The genome sizes of both strains are similar, at 2.4 MB for the oceanic strain and 2.5 MB for the coastal strain. The oceanic strain displays a greater number of DEGs (300–800) than the coastal strain (100–300) across all comparisons between treatments. The overall gene expression heatmap (Figure S2) showed that gene expression changes from warming are more consistent in the oceanic strain, whereas changes due to Fe limitation are more uniform in the coastal strain.

The top 20 most responsive DEGs for each treatment in both strains are summarised in Tables S3 and S4. They are involved in pathways of photosynthesis (*psbA, petJ, ndhD, cpeAB*), carbon and nutrient metabolism (*cysK, glnB, nirA, urtA‐D*), Fe transport (*idiA, afuC*), and heat shock response (*hsp20, clpB*). KEGG pathway enrichment analysis (Figures S3 and S4) reveals that DEGs in the oceanic strain are involved in ABC transporter and two‐component systems during Fe limitation under warming (Figure S3A, −Fe@27°C), warming under Fe limitation (Figure S3C, 27°C@‐Fe), and Fe/warming interactions (Figure S3E, −Fe + warming; Figure S3F, +Fe + warming).

### 2.3 Transcriptional Responses of Photosynthetic Pathways in Coastal and Oceanic Strains

**FIGURE 4:**
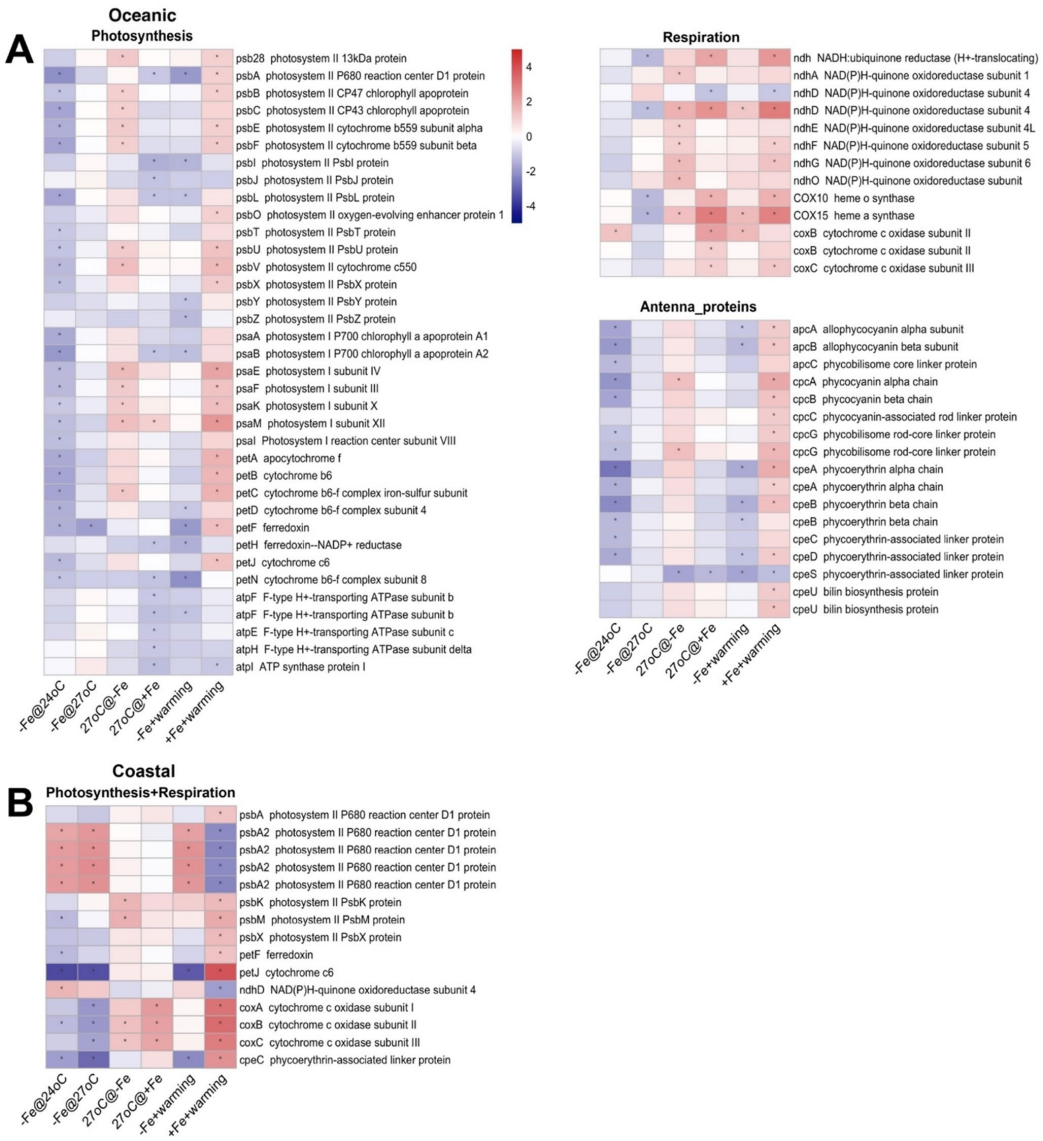
Heatmap presenting gene expression patterns of the photosynthetic pathways under different treatments in the oceanic strain (A) and the coastal strain (B). The comparison treatment columns include –Fe@24°C, –Fe@27°C, 27°C@‐Fe, 27°C@+ Fe, −Fe + warming, +Fe + warming. Detailed descriptions of each treatment comparison are given in the Methods. Each row denotes one gene with ID and annotation listed. The asterisk denotes differential expression with a fold change > 2 and an adjusted *p* < 0.05. Upregulated genes are represented in red, while downregulated genes are depicted in blue.

To investigate the distinct photosynthetic apparatus responses in both strains to Fe‐warming interactions, we compared the transcriptional responses of Fe limitation under low temperature and warming conditions (−Fe@24°C, −Fe@27°C), warming under high Fe and low Fe conditions (27°C+ Fe, 27°C@‐Fe), and Fe‐limited warming and Fe‐replete warming conditions (−Fe + warming, +Fe + warming). In the oceanic strain, at low temperature under Fe limitation (−Fe@24°C) most downregulated DEGs were involved in light harvesting and photosynthetic electron transport pathways (Figure 4A), including photosystem I (PSI) (*psaA‐I*), photosystem II (PSII) (*psbA‐F, psbL, psbT, psbUV, psbX*), cytochrome *b6f* complex (*petA‐D*), ferredoxin (*petF*), and cytochrome *c6* (*petJ*), and phycobilisome (*apcABC, cpcAB, cpcG, cpeA‐D*) genes. ATPase and oxidative phosphorylation were barely affected by Fe limitation. Similarly, under warming and Fe‐limited conditions, the oceanic strain also upregulated genes involved in photosynthesis, such as PSI (*psaEF, psaK, psaM*), PSII (*psb28, psbBC, psbEF, psbUV*), the cytochrome *b6f* complex (*petC*), and phycobiliproteins, as well as those for NADPH production (Figure 4A).

For the oceanic strain under Fe‐replete conditions, warming downregulated most DEGs related to ATP production (*atpEF, atpHI*) and photosynthesis (*psbA*, *psbI*, *psbJ*, *psbL* in PSII, *psaB* in PSI, *petH*, *petN*). However, one gene in PSI (*psaM*) was upregulated by warming under Fe‐replete conditions (Figure 4A, 27°C@+ Fe). In contrast, warming upregulated several genes related to oxidative phosphorylation (*ndhA, ndhD‐G, ndhO, COX15*) (Figure 4A, −Fe@27°C).

In the coastal strain, photosynthetic pathways were much less sensitive to Fe limitation, with only 15 DEGs in total compared to 66 DEGs in the oceanic strain (Figure 4B). However, the gene *petJ* encoding Fe‐rich cytochrome *c6* was notably downregulated at both temperatures (Figure 4B, −Fe@27°C, −Fe@24°C). Although most genes in the photosynthetic pathways were downregulated, multiple copies of *psbA2* were upregulated and *psbA1* remained unaffected under Fe limitation at both temperatures (Figure 4B, −Fe@27°C, −Fe@24°C). The DEGs under Fe limitation were similar at high and low temperatures, where *psbA2* copies were all upregulated, and *petJ* and *cpeC* were downregulated (Figure 4B, −Fe@27°C, −Fe@24°C). Surprisingly, in the coastal strain, only two genes in PSII (*psbK, psbM*) were upregulated with warming regardless of Fe conditions (Figure 4B, 27°C@+ Fe, 27°C@‐Fe), corresponding to a minimal increase in growth (Figure 4B).

In contrast, under the warming condition, gene expression in the oceanic strain was largely unaffected by Fe limitation (Figure 4A, −Fe@27°C). Rubisco (ribulose bisphosphate carboxylase) genes *rbcS* and *rbcL* were downregulated (Figure S5A, −Fe@27°C). The downregulation of the Rubisco small subunit *rbcS* at both Fe concentrations was observed (Figure S5A). Under warmer and Fe‐limited conditions, the DEGs related to carbon fixation showed no notable difference to warming in the oceanic strain (Figure S5A).

### 2.4 Comprehensive Nutrient Assimilation Network in the Oceanic Strain

To understand how nutrients were differently assimilated and utilised in both strains under Fe‐warming interactions, we compared DEGs linked to nutrient transport, regulation, and metabolism systems. The DEGs in the oceanic strain related to the ABC transporter (Figure 3C) are involved in the transport of Fe (*idiA, afuBC*), phosphonate (*phnCD*), urea (*urtABC, urtE*), manganese (Mn) (*mntAB*), biotin (*bioY*), sugars (*msmX*), polysaccharides (*kpsE, kpsM, kpsT*), and vitamin B_12_ (*bacA*). The DEGs in the two‐component system (Figure 3C) are involved in regulating P (*phoB*) and heme (*ctaA*).

**FIGURE 5:**
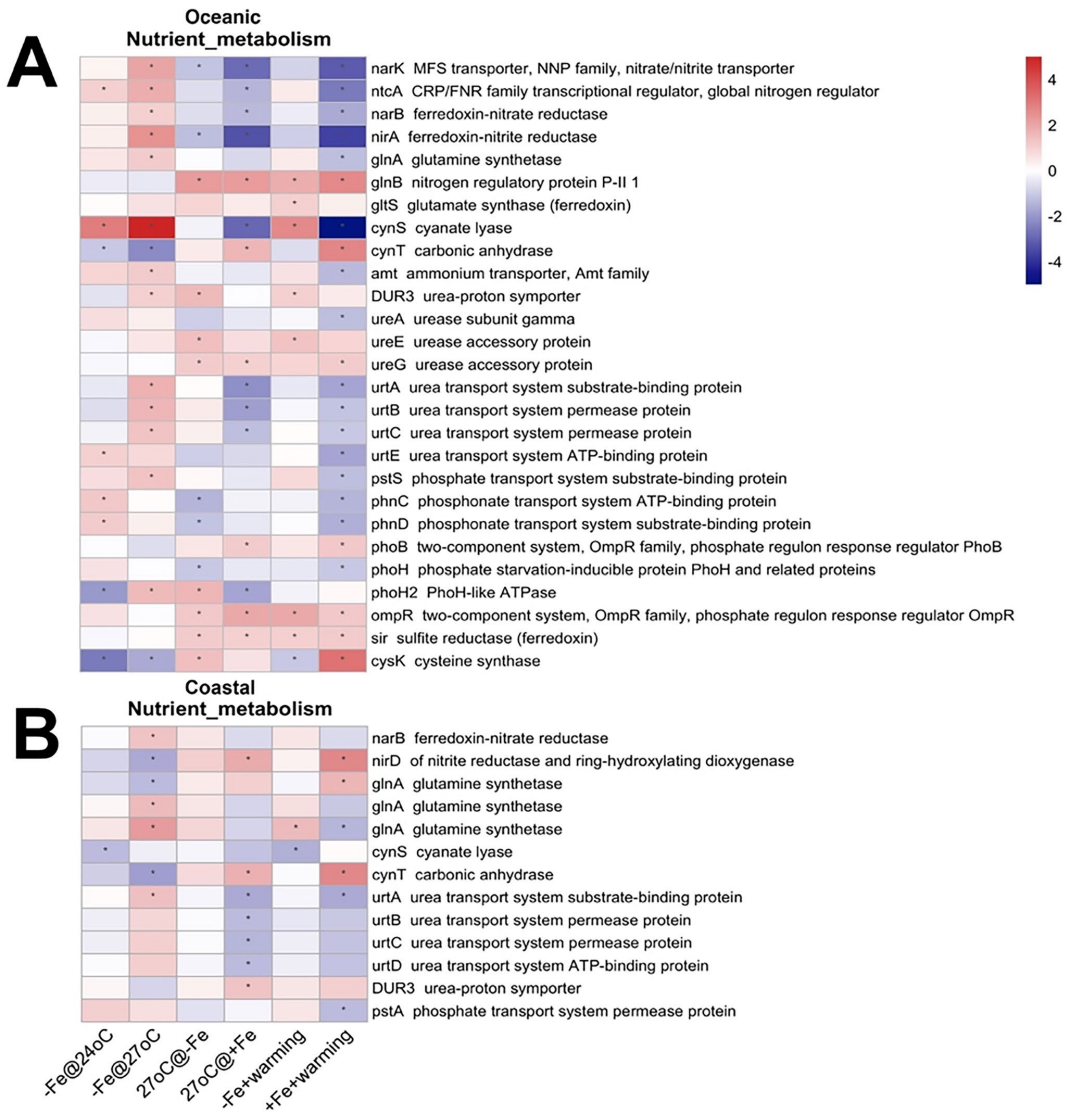
Heatmap of the gene expression patterns involved in nutrient metabolism under different treatments in the oceanic strain (A) and the coastal strain (B). The treatment columns include –Fe@24°C, –Fe@27°C, 27°C@‐Fe, 27°C@+ Fe, −Fe + warming, +Fe + warming. Red denotes upregulated DEGs, and blue denotes downregulated DEGs. Detailed descriptions of each treatment comparison are given in the Methods. Each row denotes one gene with ID and annotation listed. The asterisk denotes differential expression with a fold change > 2 and an adjusted *p* < 0.05.

Fe limitation alone upregulated most DEGs involved in nutrient metabolism in the oceanic isolate (Figure 5A, −Fe@24°C, −Fe@27°C). More DEGs, particularly those related to N and P transport systems, were upregulated under warming in Fe limited cultures, including the nitrate/nitrite transporter *narK*, global N regulator *glnA*, nitrate reductase (*narB, nirA*), ammonium transporter amt, urea transporters (*urtABC*), and the phosphate transporter *pstS* (Figure 5A). However, warming alone negatively affected nutrient metabolism regardless of Fe, with a few exceptions, including genes coding for urease accessory proteins (*ureE, ureG*) and sulfite reductase *sir* (Figure 5A, 27°C@‐Fe and 27°C@+ Fe). The PII signalling gene *glnB* was upregulated with warming alone (Figure 5A, 27°C@‐Fe, 27°C@+ Fe).

Fe limitation notably upregulated phosphonate transporters (*phnC, phnD*) at low temperatures. Warming under Fe limitation led to the downregulation of *phnC*, *phnD*, and the phosphate starvation‐inducible gene *phoH*, while phosphate regulators *phoB* were upregulated. In contrast, phosphate transporter genes were not significantly affected by either warming or Fe limitation. Instead, Fe limitation under warming conditions in the coastal strain upregulated ferredoxin‐nitrate reductase *narB*, glutamine synthetase *glnA*, and a urea transport gene *urtA* (Figure 5B, −Fe@27°C). More DEGs were observed when Fe limitation was induced under warming conditions (Figure 5B, −Fe@27°C) than at low temperatures (Figure 5B, −Fe@24°C). Warming under Fe‐replete conditions (27°C@+ Fe) upregulated nitrite reductase *nirD* while downregulating all the urea transport genes *urtA‐D*.

### 2.5 Transcriptional Responses for Fe Transport, Regulation, and Storage

**FIGURE 6:**
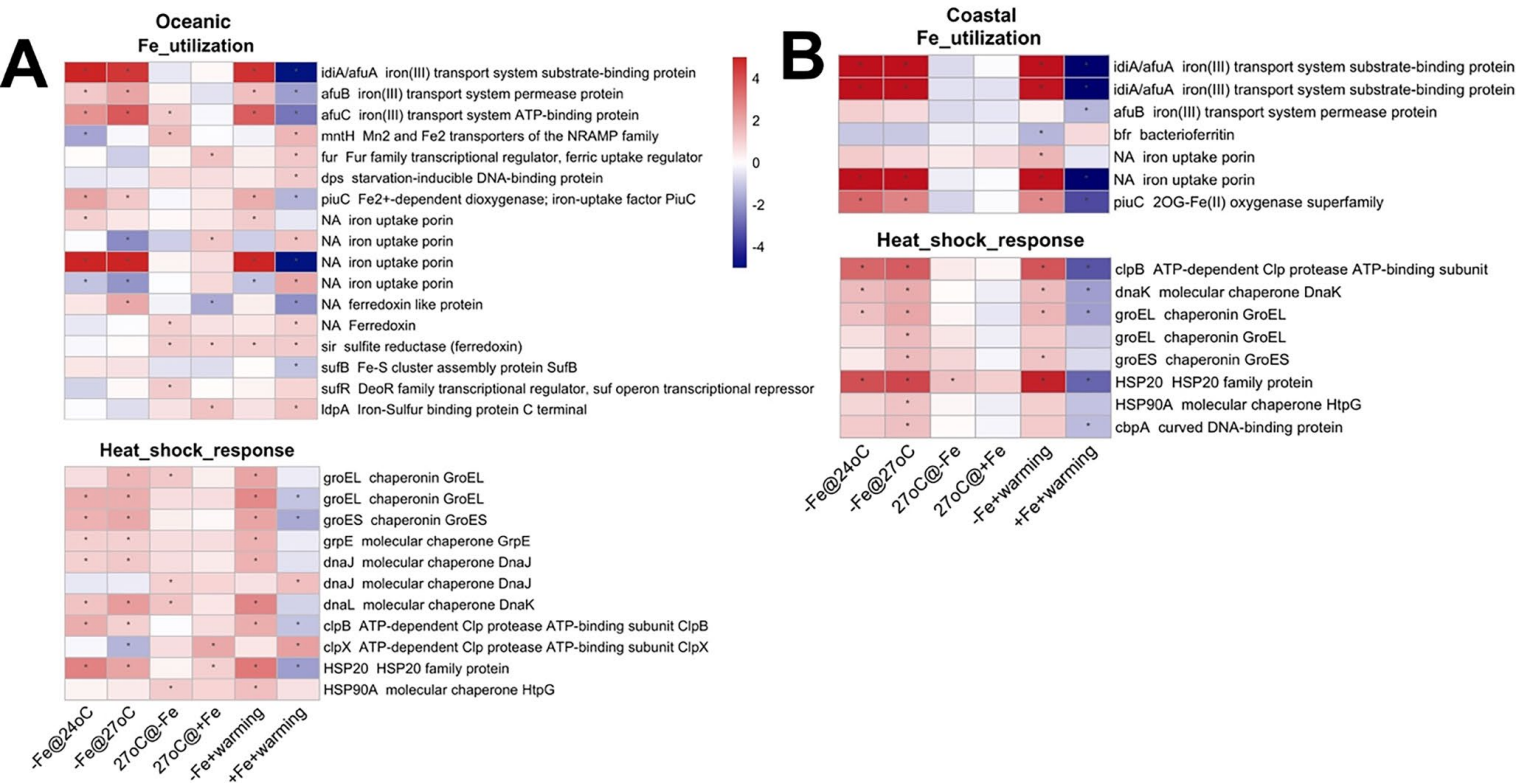
The heatmap presents the gene expression patterns involved in Fe utilisation and heat shock response under different treatments in the oceanic strain (A) and the coastal strain (B). The treatment columns include –Fe@24°C, –Fe@27°C, 27°C@‐Fe, 27°C@+ Fe, −Fe + warming, +Fe + warming. Red denotes upregulated DEGs, and blue denotes downregulated DEGs. Detailed descriptions of each treatment comparison are given in the Methods. Each row denotes one gene with ID and annotation listed. The asterisk denotes differential expression with a fold change > 2 and an adjusted *p* < 0.05.

To explore how Fe‐related metabolism responded under different Fe‐warming conditions, we characterised the DEGs involved in Fe transport, regulatory, and storage systems in the two isolates. Both strains exhibited a significant upregulation of Fe stress biomarkers such as *idiA*, *afuB*, and *afuC* under Fe limitation regardless of temperature (Figure 6A,B). Fe porin genes were significantly expressed under various Fe‐warming conditions (Figure 6A,B).

Warming upregulated genes like *afuC* (27°C@‐Fe), *fur* (27°C@+ Fe), and Fe‐S cluster regulator *sufR* (27°C@‐Fe) and *idpA* (27°C@+ Fe) in the oceanic strain (Figure 6A). The *suf* operon transcriptional repressor *sufR* was downregulated with warming under Fe limitation. The *dps* gene (encoding the DNA binding protein in starved cells) was upregulated under +Fe + warming conditions (Figure 6A). Similarly, bacterioferritin in the coastal strain was downregulated under −Fe + warming conditions (Figure 6B). In the coastal strain, the Fe^3+^ transporter *idiA*, Fe uptake porins, and the Fe uptake factor *piuC* were all responsive to warming (Figure 6B).

Heat shock genes (*groEL/groES* (*hsp60/hsp10*), *dnaK/dnaJ/grpE* (*hsp70/hsp40/nucleotide exchange factor*), *htpG/hsp90*, and *clpB/hsp100*) were more responsive to Fe limitation than to warming in both strains, but were especially prominent under the −Fe+ warming condition, indicating an important role for Fe and warming interactive stress protection by these chaperone proteins (Figure 6, −Fe vs. + Fe@24°C, −Fe vs. + Fe@27°C). Warming alone led to the upregulation of antioxidant genes in the oceanic strain, such as those coding for methionine sulfoxide reductase and thioredoxins. The oceanic strain upregulated SOD1 (Cu‐Zn family superoxide dismutase) with warming when Fe was limiting (Figure S6A).

### 2.6 Fe‐Warming Interactions in Both Strains

In addition to examining the individual effects of Fe concentrations and temperatures, we explored their interactive effects (Fe‐warming interactions) to determine whether there are non‐linear effects of changes in both factors simultaneously. The interactions between Fe and warming have a more pronounced effect on gene expression than either factor alone in both strains (Figure 3A). In the oceanic strain, many gene expression patterns in photosynthesis were similar between −Fe@27°C and 27°C@‐Fe (Figure 4A). The DEGs involved in oxidative phosphorylation were upregulated under the −Fe + warming comparison treatment, consistent with warming at Fe limitation but opposite to Fe limitation at warming comparison treatments. These indicate a predominant impact of temperature. However, antenna proteins were generally unaffected by either Fe limitation or warming alone, yet were downregulated under −Fe + warming (‐Fe@27°C vs. + Fe@24°C; Figure 4A). Furthermore, the DEG patterns under −Fe + warming were consistent with 27°C@‐Fe (Figure 5), and the patterns under +Fe + warming conditions were similar to 27°C@+Fe, indicating warming plays an important role in nutrient metabolism in the oceanic strain. Most Fe utilisation and heat shock genes showed opposite trends under +Fe + warming and −Fe + warming conditions (Figure 6A), indicating that these genes are more affected by Fe concentrations.

**FIGURE 7:**
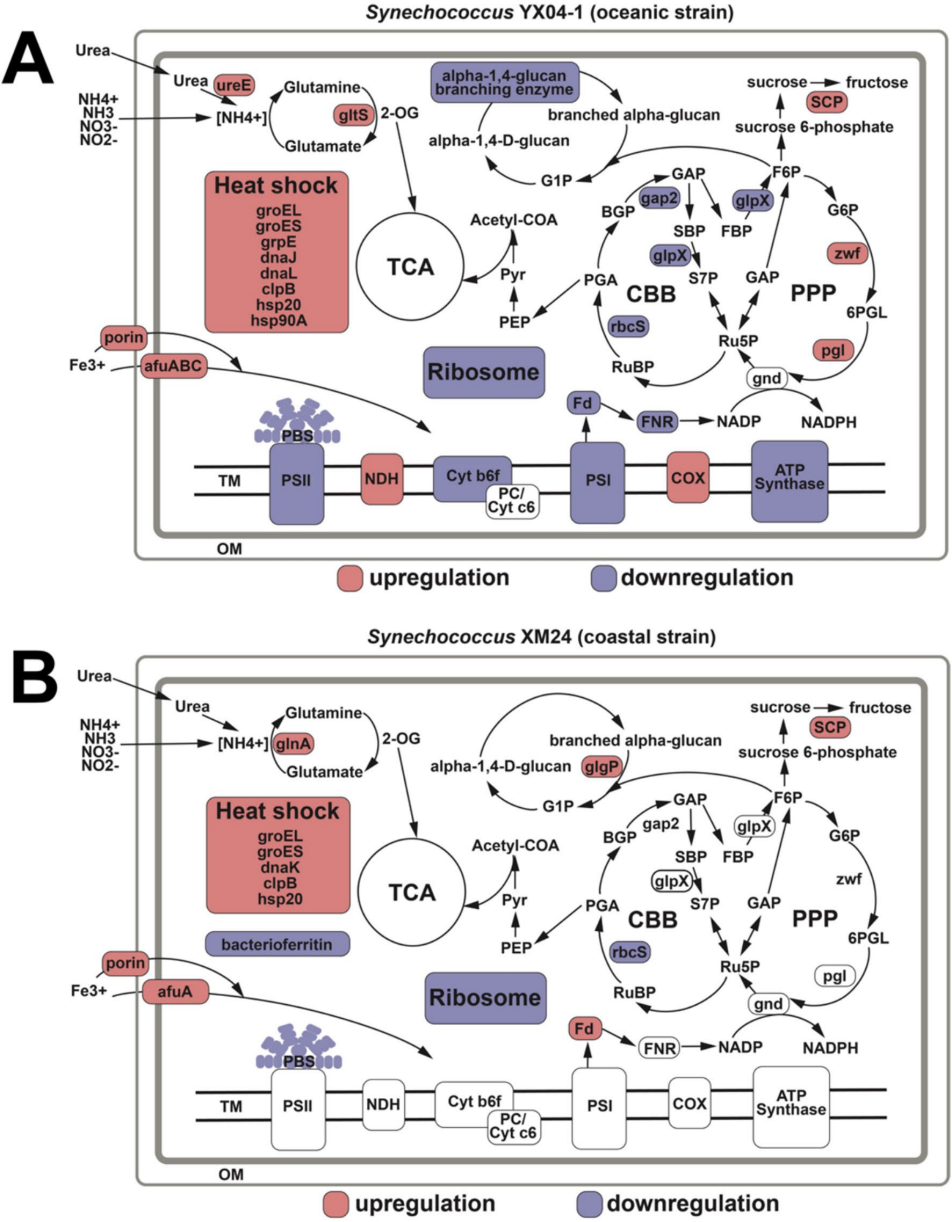
Summary figure depicting gene expression under –Fe + warming interactive conditions in the oceanic strain (A) and the coastal strain (B). Red denotes gene upregulation and blue denotes gene downregulation. 2‐OG, 2‐oxoglutarate; 6PGL, 6‐Phosphogluconolactone; BGP, 1,3‐bisphosphoglycerate; Cyt b_6_f, cytochrome b_6_f complex; F6P, fructose 6‐phosphate; FBP, fructose‐1,6‐bisphosphate; Fd, ferredoxin; FNR, ferredoxin‐NADP(+) oxidoreductase; G1P, glucose 1‐phosphate; G6P, glucose‐6‐phosphate; GAP, glyceraldehyde‐3‐phosphate; NDH, NADH dehydrogenase; OM, outer membrane; PBS, phycobilisomes; PC/Cyt c_6_, plastocyanin/cytochrome c_6_; PEP, phosphoenolpyruvate; PGA, 3‐phosphoglycerate; PSI, photosystem I; PSII, photosystem II; Pyr, pyruvate; Ru5P‐ribulose‐5‐phosphate; RuBP, ribulose‐1,5‐bisphosphate; S7P, sedoheptulose‐7‐phosphate; SBP, Sedoheptulose 1,7 bisphosphate; TM, thylakoid membrane.

In comparison, the coastal strain showed gene expression patterns under −Fe + warming that were similar to those under Fe limitation at both temperatures, and patterns under +Fe + warming are opposite to those under‐Fe at 27°C (Figure 4B). This indicates that Fe plays a predominant role in photosynthetic activities in the coastal strain. Unlike in the oceanic strain, 27°C at‐Fe and −Fe + warming had minimal effect on nutrient metabolism in the coastal strain, indicating the nuanced effect of temperature at low Fe on the coastal strain. Furthermore, warming had no effect on Fe utilisation and only one heat shock gene, *hsp20*, was upregulated at 27°C at‐Fe (Figure 6B), indicating that these pathways were regulated by Fe concentrations. In summary, under −Fe + warming conditions, the oceanic strain regulated pathways including photosynthesis, nutrient metabolism, heat shock, and Fe transport (Figure 7A), and the coastal strain regulated similar pathways but with many fewer genes (Figure 7B).

## 3 Discussion

### 3.1 Differential Sensitivity to Fe‐Warming Interactions

We explored how the sensitivity of the two isolates can influence their acclimation to environmental changes. The oceanic strain is more responsive to Fe‐warming interactions and may experience greater selective pressure from both factors in oligotrophic regions. Both Fe and temperature changes significantly affect physiology and central metabolism, including photosynthesis and nutrient assimilation, which aligns with previous studies in marine *Synechococcus* (Liu and Qiu 2012; Mackey et al. 2015).

In the oceanic strain, ABC transporters and two‐component regulatory systems were significantly enriched under Fe‐warming interactions, including those for Fe, nutrients, Mn, and polysaccharides. This indicates that under future warming in oligotrophic areas, the oceanic strain may have more ability to utilise various resources and sense and respond to environmental changes in the ocean.

### 3.2 Distinct Photosynthetic Strategies in Response to Fe‐Warming Interactions

We found that the two isolates employ distinct photosynthetic strategies to cope with Fe‐warming interactions. Physiological and transcriptomic data highlighted how each isolate adjusts phycobilisome organisation, electron flow, and carbon fixation under different Fe‐warming scenarios. Under Fe‐limited conditions at low temperature, the oceanic strain reduces the synthesis of Fe‐rich photosynthetic components, particularly PSII (2 Fe atoms), PSI (12 Fe atoms), cytochrome *b6f* (6 Fe atoms), and ferredoxin, to conserve Fe and minimise oxidative stress from impaired electron transport (Blanco‐Ameijeiras et al. 2017). Fe limitation also significantly reduced the expression of phycobiliprotein genes in the oceanic *Synechococcus*. Adjusting phycobilisome composition (including allophycocyanin, phycocyanin, and phycoerythrin) to better harvest green and blue wavelengths may be beneficial during deep heat waves, when warming conditions can extend into the lower euphotic zone (Chen et al. 2022; Santana‐Falcón and Séférian 2022; Fragkopoulou et al. 2023).

Under Fe‐replete conditions, warming had little effect on the growth rates and photosynthesis in the oceanic strain. The oceanic strain may employ state transitions to modulate the distribution of energy absorbed by phycobilisomes. Under Fe‐limited conditions, warming may induce state 1, where the phycobilisome associates with PSII (Mackey et al. 2013). Under Fe‐replete conditions, warming may cause the oceanic strain to shift to state 2, where the PBS associates with PSI. The oceanic *Synechococcus* may utilise both linear electron flow and cyclic electron flow around PSI to generate ATP (Behrenfeld et al. 2008). These responses may indicate a strategy to balance the allocation of photosynthetic resources (ATP and NADPH) among cellular processes to maximise growth and metabolism. However, these proposed state transitions were based on the interpretations of the transcriptomic responses of photosynthetic pathways, and direct experimental validation will be needed in future studies.

RuBisCO, the key enzyme for carbon fixation, was downregulated with warming. This may be compensated by increasing carboxysome numbers while reducing the amount of RuBisCO per carboxysome (Dedman et al. 2023). Cytochrome c oxidase (respiratory terminal oxidase) was upregulated, indicating a greater reliance on pathways by which electrons flow from PSII to a terminal oxidase, utilising O_2_ as the electron acceptor (Blanco‐Ameijeiras et al. 2018). This may explain the higher carbon fixation rates observed with warming, despite no significant difference in growth rates.

The coastal strain has a higher Fe quota, likely due to requirements in photosynthesis, particularly PSI. D1 genes in PSII were highly expressed, which require less Fe than PSI. The coastal strain has a higher turnover rate of D1 protein isoforms between D1:1 (coding by *psbA*) and D1:2 (coded by *psbAII*). D1:2 appears to be more stress‐resistant than D1:1 when excitation pressure on PSII increases due to light or temperature stress (Sane et al. 2002). Downregulation of cytochrome c oxidase (respiratory terminal oxidase) by warming leads to transient reduction in photosynthetic oxygen evolution during the period of D1:1/D1:2 exchange. Therefore, the coastal strain may upregulate PSII genes without greatly increasing Fe demand under Fe‐limited conditions to optimise energy use.

Hence, the oceanic and the coastal *Synechococcus* use distinct strategies to adapt photosynthesis to Fe‐warming stress. These include potential state transitions of phycobilisomes between PSI and PSII to facilitate phycobilisome energy excitation (Bhatti et al. 2021), reducing oxidative stress, adjusting ATP and NADPH production to maximise metabolism in the oceanic strain, and the fast turnover of D1 isoforms in PSII (Ahlgren and Rocap 2006) in the coastal strain.

### 3.3 Selective Nutrient Acquisition Preferences in the Two Strains

We investigated how each isolate shapes its preferences for organic versus inorganic nutrients under changing Fe availabilities and temperatures. The oceanic strain under warming at both Fe concentrations seemed to rely more on organic N sources that require less Fe to metabolise (Sharpe et al. 2023). These alternative N acquisition strategies in the oceanic strain with increasing temperature may help it to better deal with N scarcity in oligotrophic areas. At low temperature, Fe limitation by itself also increases the gene expression of urea and phosphonate transport systems, again indicating that N and P sources were redirected to organic forms. This may be because nitrate reductase and nitrite reductase both require Fe (Flynn and Hipkin 1999). This correlated with the downregulation of nitrogen regulatory genes *glnB* and *ntcA* by warming when Fe is replete. PII proteins coded by *glnB* are signal transduction proteins that regulate the activities of both glutamine synthetase and the global nitrogen transcriptional factors (Huergo et al. 2013). *ntcA* is a global transcriptional regulator in *Synechococcus* that responds to various N sources. When low concentrations of 2‐oxoglutarate (2‐OG) are sensed by the signal transduction protein PII, *ntcA* is activated to increase nitrate/nitrite transport and assimilation (Tanigawa et al. 2002).

Conversely, the coastal strain showed an opposite trend on N source. It indicated a preference for inorganic N sources, including nitrate and nitrite, possibly due to increased Fe and inorganic nutrient availability in coastal and estuarine areas than in oligotrophic areas. N availability plays a more critical role in shaping Fe limitation strategies than other nutrients like phosphorus (Mackey et al. 2015). Hence, Fe‐warming interactions may significantly influence N:P ratios of marine *Synechococcus* and further impact marine carbon and nitrogen cycles.

### 3.4 Ocean Warming Favours the Oceanic Strain Under Fe‐Limited Conditions

We found that warming benefits the oceanic strain for both physiological and transcriptomic responses, such as photosynthesis, metal transport, nutrient uptake, and translational machinery. Warming increased the growth rates in the oceanic strain under Fe‐limited conditions, with most photosynthesis genes upregulated. Fe (III) and manganese transport were upregulated. These important metal cofactors are involved in the water‐splitting complex of PSII (the Mn_4_CaO_5_‐cluster complex) (Sauer and Yachandra 2002), thus playing key roles in O_2_ evolution.

Uptake genes for nitrate, nitrite, and urea, genes coding glutamate synthetase, and phosphate transport genes were all upregulated by warming under Fe‐limited conditions, indicating increased nutrient utilisation even under Fe stress. Under warming conditions, Fe limitation had little effect on photosynthesis pathways in the oceanic strain, consistent with significantly higher growth rates under Fe limitation with warming compared to lower temperature. This suggests that warming may partly alleviate Fe limitation in the oceanic strain and induce higher growth under Fe‐limited conditions. Increased Fe(III) transport under warming further supports enhanced Fe uptake.

Under warming conditions, ribosomes which are rich in phosphorus were mostly downregulated with increasing temperature (Figure S7). Ribosomes are essential for protein synthesis and are temperature‐sensitive (Li et al. 2019), so their downregulation may result from increased translation efficiency, leading to more efficient protein synthesis with fewer ribosomes (Dedman et al. 2023). This suggests that translational machinery, rather than transcription or transcript stability, is the primary target of heat stress (Allakhverdiev et al. 2007). Our study indicates the molecular mechanisms favouring oceanic *Synechococcus* under warming and Fe‐limited conditions include utilising low Fe demand organic N forms and diverting substantial resources to photosynthesis to induce faster growth. This may provide a major advantage for the oceanic strain to thrive in warming oligotrophic oceans.

### 3.5 Multi‐Tiered Fe Acquisition Strategies in Both Strains

We explored how the two isolates employ diverse Fe acquisition and storage strategies to meet the Fe demand for essential metabolic processes. The coastal strain exhibited a higher Fe quota than the oceanic strain, which is a common strategy that enables luxury Fe uptake and better adaptation to fluctuating Fe levels (Twining et al. 2021). Coastal waters generally have higher Fe concentrations than the open ocean (Mackey et al. 2015), and opportunistic storage may contribute to the coastal strain's tolerance of episodic Fe limitation.

However, high internal Fe quotas can expose cells to oxidative stress because free Fe can participate in Fenton reactions, generating harmful hydroxyl radicals (Shcolnick et al. 2009). To mitigate oxidative stress, we found that the coastal strain utilises bacterioferritin, while the oceanic strain employs a ferritin‐like *dps* gene for Fe storage. Bacterioferritin oxidises Fe(II) to Fe(III), storing Fe(III) as Fe oxide in the complex and generating hydrogen peroxide to reduce oxidative stress (Shcolnick et al. 2009). The *dps* gene helps protect DNA by sequestering Fe, mitigating photooxidative damage under stressed conditions in the oceanic strain (Castruita et al. 2006). The internal recycling mechanisms help maintain a stable Fe supply for cellular metabolic processes under Fe–warming interactions.

Both strains showed a common mechanism of Fe (III) acquisition (Rivers et al. 2009). Furthermore, both strains exhibited differential expression of porin genes under different Fe–warming interactions. Porins are transmembrane proteins that form channels in the outer membrane, allowing passive diffusion of small molecules like carbohydrates, amino acids, and Fe(II) (Qiu et al. 2018). The oceanic strain specifically utilised the transcriptional regulator *fur*. Fur acts as a transcriptional repressor when bound to intracellular Fe (II) (Mackey et al. 2015). Our results revealed a connection between the Fe and N regulatory networks involving *fur* and *ntcA*. These networks regulate Fe transport for metalloenzymes involved in N assimilation, such as nitrate reductase (López‐Gomollón et al. 2007), potentially influencing Fe and N cycling in the open ocean.

In general, both the oceanic and coastal strains have developed various strategies to cope with Fe limitation, including using high‐affinity transporters to increase Fe uptake, reducing Fe quota, recycling internal Fe pools, storing Fe in ferritin, and reducing oxidative damage resulting from Fe limitation, all of which can further influence Fe bioavailability and distribution in the ocean.

### 3.6 General Heat Shock and Antioxidative Responses in Both Strains

Defensive molecular strategies were documented in the two isolates to maintain protein activities and cellular function under stressed conditions. Molecular chaperones assist in protein folding, assembly, and stability under Fe‐warming conditions (Rajaram et al. 2014; Mishra and Grover 2016). The *hsp90A* gene coding for HtpG protein is a general stress protein that is essential for thermotolerance and can prevent the aggregation of phycobiliproteins, thereby protecting the photosynthetic apparatus (Rajaram et al. 2014). Continuous overexpression of *clpB* and the use of a strong constitutive promoter (*psbA2*) allow for an immediate response to heat shock (Gonzalez‐Esquer and Vermaas 2013) in the coastal strain. The *dnaK* chaperone gene is important for regulating and stabilising the *psbAII* in the coastal strain. *clpB* and *dnaK* genes showed upregulation under Fe limitation at both temperatures and downregulation under high Fe warming interactions, which suggests their cooperation to enhance tolerance to both warming (Gonzalez‐Esquer and Vermaas 2013) and Fe stress.

Warming can enhance enzyme activities and lead to more ROS production under heat stress, while Fe limitation can lead to increased oxidative stress due to the breakdown of Fe‐S clusters in proteins (Shafiee et al. 2022). Only the oceanic strain adjusted gene expression of the Cu‐Zn family superoxide dismutase, suggesting greater utilisation of copper. Although the heat shock genes in both strains responded to Fe limitation similarly, the oceanic strain responded to warming alone with more upregulated genes. This suggests that there are more options for the oceanic strain to deal with future warming.

### 3.7 Comparative Transcriptomic and Proteomic Responses to Fe‐Warming Interactions

Schiksnis et al. (2024) used proteomic approaches to explore *Synechococcus* responses to Fe and warming interactions in these same experiments. Hence, we compared the alignment between mRNA expression and protein abundance in the two isolates. Similarly, they showed that Fe changes had a greater impact than temperature changes on both physiology and protein abundance in key metabolic pathways such as photosynthesis, antioxidation, and translation. Moreover, as with the transcriptomes presented here, the proteome of the oceanic strain was more responsive to Fe–warming interactions than the coastal strain (Schiksnis et al. 2024).

Comparing the proteomic and transcriptomic responses in this study, we found that the transcriptional regulatory network, including *fur* and the two‐component systems for nitrogen, phosphorus, and metal acquisition in the oceanic strain, ultimately helped adjust the proteomic allocation of Fe metalloenzymes involved in photosynthesis and antioxidative responses. General responses related to antioxidation, translation efficiency, and heat shock were significantly expressed in both transcriptomic and proteomic analyses. This indicates that mRNA and proteins involved in environmental stress were closely correlated, which is not necessarily always the case in comparative studies in cyanobacteria (Walworth et al. 2022). Both this study and the proteomic responses reported by Schiksnis et al. (2024) provide insights into the metabolic networks of transcripts and proteins associated with Fe‐warming interactions. However, the detailed mechanisms behind the correlations between transcriptome and proteome may need further consideration, including basal expression levels across different pathways under environmental stress, rates of transcript and protein production and turnover, and post‐translational modifications (Walworth et al. 2022).

## 4 Conclusions

The two marine *Synechococcus* strains from coastal and offshore clades employed different strategies in response to simultaneous Fe limitation and warming. These included distinct photosynthetic and carbon fixation strategies to maximise energy production, selective preferences between inorganic and organic forms of nutrients, divergent regulatory networks employed for Fe and nutrient reallocation under different Fe–warming interactions, and different ways to increase translational efficiency and reduce oxidative and osmotic stress caused from Fe–warming interactions. This could further influence the proteomic responses of the two strains to adjust photosynthesis and oxidative stress in response to Fe–warming interactions, as indicated by Schiksnis et al. (2024). The effect of temperature on marine *Synechococcus* is dependent on the Fe concentrations experienced by the organism, and vice versa. The oceanic *Synechococcus* clade II appears to be especially well adapted to deal with the combination of Fe and thermal stress, and may thus outcompete many other open ocean phytoplankton groups under changing ocean conditions (Ahlgren and Rocap 2012).

Our study indicates that Fe limitation and global warming interact in complex and non‐linear ways in marine *Synechococcus*, resulting in co‐limitation, synergisms, and antagonisms that can diminish or amplify the impacts of climate change. The combination of both factors may not ultimately result in increased productivity. This emphasises the need for multivariate research approaches to better predict the impacts of global climate change on marine ecosystems. Further laboratory and field studies may explore how *Synechococcus* clades from different areas acclimate to interactions between warming and other key environmental change factors, including irradiance, salinity, oxidative stress, and nutrient availability. Only then can we begin to elucidate the full spectrum of potential *Synechococcus* adaptive responses to a changing ocean across diverse regimes and lineages.

## 5 Experimental Procedures

### 5.1 Culturing Conditions and Experimental Design

*Synechococcus* strains XM24 and YX04‐1 were isolated from the coastal and offshore regions of the South China Sea, respectively (Schiksnis et al. 2024; Zheng et al. 2018). Phylogenetic analysis classified them into subcluster 5.2 clade CB5 (XM24) and subcluster 5.1 clade II (YX04‐1). The cultures were grown in Aquil medium using trace metal clean artificial seawater (Sunda et al. 2005). The experiments were conducted using a matrix of two Fe concentrations (2 and 250 nM) and two temperatures (24°C and 27°C) under a 12:12 dark/light cool‐white fluorescent light at ~30 μmol quanta m^−2^ s^−1^. A semi‐continuous approach (Yang et al. 2021) was employed to grow the cultures under each treatment condition for at least 2 months (12 generations or more) prior to measuring physiological responses and collecting RNA samples. Physiological measurements included growth rates, Fe quota, carbon fixation rates, and particulate organic carbon (POC) and particulate organic nitrogen (PON). RNA samples were flash‐frozen and stored in liquid nitrogen until extraction and sequencing. Three replicates were collected for all parameters under each iron/warming condition.

Transcriptomic analysis was conducted to identify differentially expressed genes across different treatments. To compare and contrast the responses of both isolates to warming and Fe limitation individually and in combination, six‐treatment comparisons were conducted: (1) −**Fe@27°C**: Fe‐limited versus Fe‐replete at 27°C; (2) −**Fe@24°C**: Fe‐limited versus Fe‐replete at 24°C; (3) −**Fe/warming interaction (−Fe + warming)**: 27°C Fe‐limited versus 24°C Fe‐replete; (4) **27°C@+ Fe**: 27°C Fe‐replete versus 24°C Fe‐replete; (5) **27C@‐Fe**: 27°C Fe‐limited versus 24°C Fe‐limited; (6) **+ Fe/warming interaction (+Fe + warming)**: Fe‐replete at 27°C versus Fe‐limited at 24°C (Table S1). In our study, “warming” strictly refers to the comparison between 24°C (low temperature) and 27°C (high temperature)—both within the natural thermal range of these strains—and does not imply novel temperature increases outside that range.

### 5.2 Growth Rates, Carbon Fixation Rates and Elemental Stoichiometry

The cultures were isolated at ~25°C–28°C, which is the mean surface temperature in the South China Sea (Yu et al. 2019), and hence 24°C and 27°C were used in the experiments to bracket this ambient temperature. The cultures were diluted every other day based on in vivo fluorescence readings. Cell growth rates were determined using the equation *μ* = ln(N/N_0_)/(t‐t_0_), where N represented the final cellular in vivo fluorescence at time t, and N_0_ represented the initial in vivo fluorescence at time t_0_.

Carbon fixation rates were assessed using ^14^C‐labelled bicarbonate. 50 mL samples from each bottle were incubated with ^14^C for 3 h, and then filtered onto GF/F membranes. Subsequently, ^14^C radioactivity of the filters was measured using a Beckman System 6500 liquid scintillation counter, converted to carbon fixation rates, and normalised to particulate organic carbon concentration (Fu et al. 2008). For particulate organic carbon and nitrogen (POC and PON) determinations, cultures were filtered onto pre‐combusted glass microfiber filters, which were dried in an oven at ~60°C and analysed using a Costech Elemental Analyser calibrated with methionine and acetanilide (Fu et al. 2008).

### 5.3 Fe Quota Measurements

To obtain iron quota results, cell samples were filtered, digested, and analysed by mass spectrometry following published methods (Yang et al. 2021; Hawco et al. 2021). Briefly, cultures were filtered onto acid‐washed 0.2 μm Supor polyethersulfone filters and rinsed with an oxalate reagent to eliminate extracellular trace metals (Kustka et al. 2004). The filters were digested with 5 mL of 50% nitric acid (HNO_3_) at 95°C for 5 days in 30 mL perfluoroalkoxy vials (Savillex). After removing the filters and drying the samples at 100°C, they were resolubilised in 200 μL of 1:1 concentrated HNO_3_ and hydrochloric acid (HCl), sealed and heated for approximately 2–3 h. The samples were dried down again and then resuspended in 5 mL of 0.1 M distilled HNO_3_ for Fe and P concentration analysis using a Thermo Scientific Element2 inductively coupled plasma mass spectrometry (ICP‐MS). The cellular Fe quota was represented by the Fe concentration normalised to P content, and to POC (Kustka et al. 2003).

### 5.4 Statistical Analysis

The significance of the physiological parameters between Fe and temperature changes was assessed via two‐way ANOVA and a Tukey multiple comparison test at *p* < 0.05 in Graphpad prism v9.5.1, including growth rates, Fe quotas, and carbon fixation rates.

### 5.5 RNA‐Sequencing and Transcriptomic Analysis

Molecular samples were collected using polycarbonate filters, flash‐frozen in liquid nitrogen, and stored in liquid nitrogen until sequencing. RNA was extracted using the Direct‐zol RNA Miniprep Kit (Zymo Research) following the manuals. The transcriptomes were paired‐end sequenced by Illumina HiSeq/Miseq with quality control, library construction, and sequencing procedures.

After sequencing, adapter sequences and low‐quality bases were trimmed from raw reads in fastq format using Atropos. The quality of the trimmed reads was confirmed using FastQC v0.11.2 (Andrews 2010). Ribosomal RNAs were removed using SortMeRNA v2.0 (Kopylova et al. 2012) with default parameters and the remaining clean non‐rRNA reads were aligned to *Synechococcus* reference genomes using BWA MEM v0.7.12 (Li 2013) with default parameters. The number of reads aligned to each gene feature was counted using featureCounts v1.6.0 (Liao et al. 2014) and differentially expressed genes were identified using DESeq2 v1.24.0 (Love et al. 2014) with specified cutoffs for log2 fold change > 1 and adjusted *p* < 0.05. TMM‐normalised read counts in counts per million (CPM) were also calculated using edgeR v3.26.8 (Robinson et al. 2010) to compare gene expression across treatments. KEGG functional enrichment analysis was performed using clusterProfiler v3.12 (Yu et al. 2012). Heatmaps were generated using the pheatmap v1.0.12 and all other figures were generated using ggplot2 v3.3.6 in R studio.

## Author Contributions

**Ran Duan:** conceptualization, methodology, data curation, investigation, formal analysis, visualization, writing – original draft, writing – review and editing. **Min Xu:** conceptualization, methodology, data curation. **Xiaopeng Bian:** conceptualization, methodology, data curation. **Conner Y. Kojima:** methodology, visualization. **Shengwei Hou:** methodology, visualization. **Qiang Zheng:** conceptualization, funding acquisition, supervision. **Seth G. John:** conceptualization, funding acquisition, supervision. **David A. Hutchins:** conceptualization, funding acquisition, project administration, writing – review and editing, supervision. **Fei‐Xue Fu:** conceptualization, methodology, data curation, supervision, project administration, funding acquisition, writing – review and editing.

## Conflicts of Interest

The authors declare no conflicts of interest.

## Supporting information


**Data S1.** Supporting Information.

**Data S2.** Supporting Information.

**Data S3.** Supporting Information.

**Data S4.** Supporting Information.

## Data Availability

The transcriptome sequences of both coastal and oceanic *Synechococcus* have been uploaded to NCBI Sequence Read Archive under the Bioproject ID PRJNA1101000, and physiology data are archived at BCO‐DMO (https://www.bco‐dmo.org/project/786679). All the scripts and codes for data analysis and visualisation are available at GitHub (https://github.com/duanrandr/Syn_transcriptome_Fe_warming).
