# Supplementary material for: Contrasting Responses of Oceanic and Coastal Synechococcus to Iron Limitation and Warming Interactions
Source: Environ Microbiol Rep. 2025 Jul 18;17(4):e70158. doi: 10.1111/1758-2229.70158 (PMC12274634; doi:10.1111/1758-2229.70158)
Supplement: Supplementary file 1 — Data S1. Supporting Information. [file EMI4-17-e70158-s002.docx]

**Supplementary Materials**

**This PDF file includes:**

Figs. S1 to S7

Table S1

**Other Supplementary Materials for this manuscript include the following:**

Tables S2 to S4


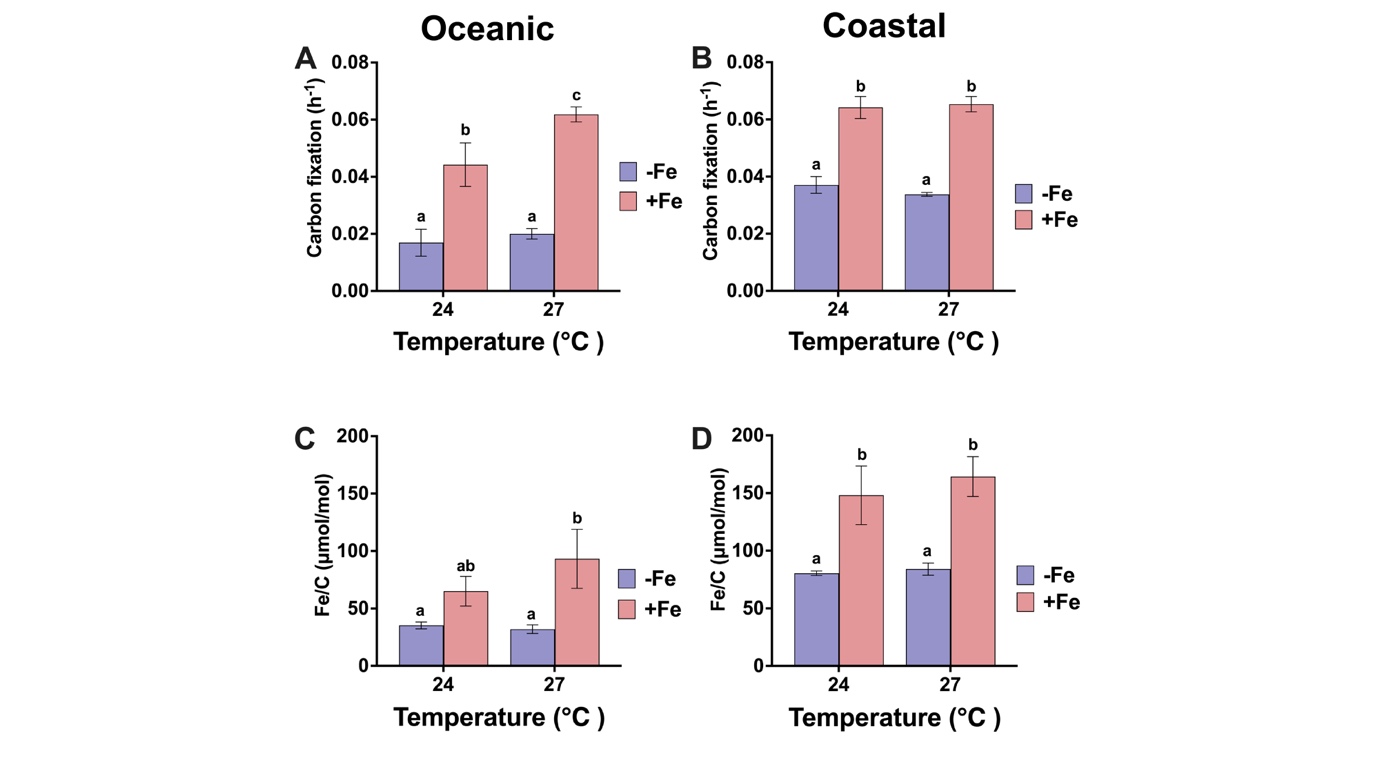


**Fig. S1.** The carbon fixation rates (h^-1^) for the oceanic strain (A) and the coastal strain (B), as well as the Fe quota indicated by the Fe to C ratio (mmol/mol) for the oceanic strain (C) and the coastal strain (D) are illustrated. The x-axis represents the different incubation temperatures used in the experiment. Error bars are standard deviations of triplicates. Mean values that do not share the same letter are significantly different from one another with p-values < 0.05 (tested by two-way ANOVA).

**
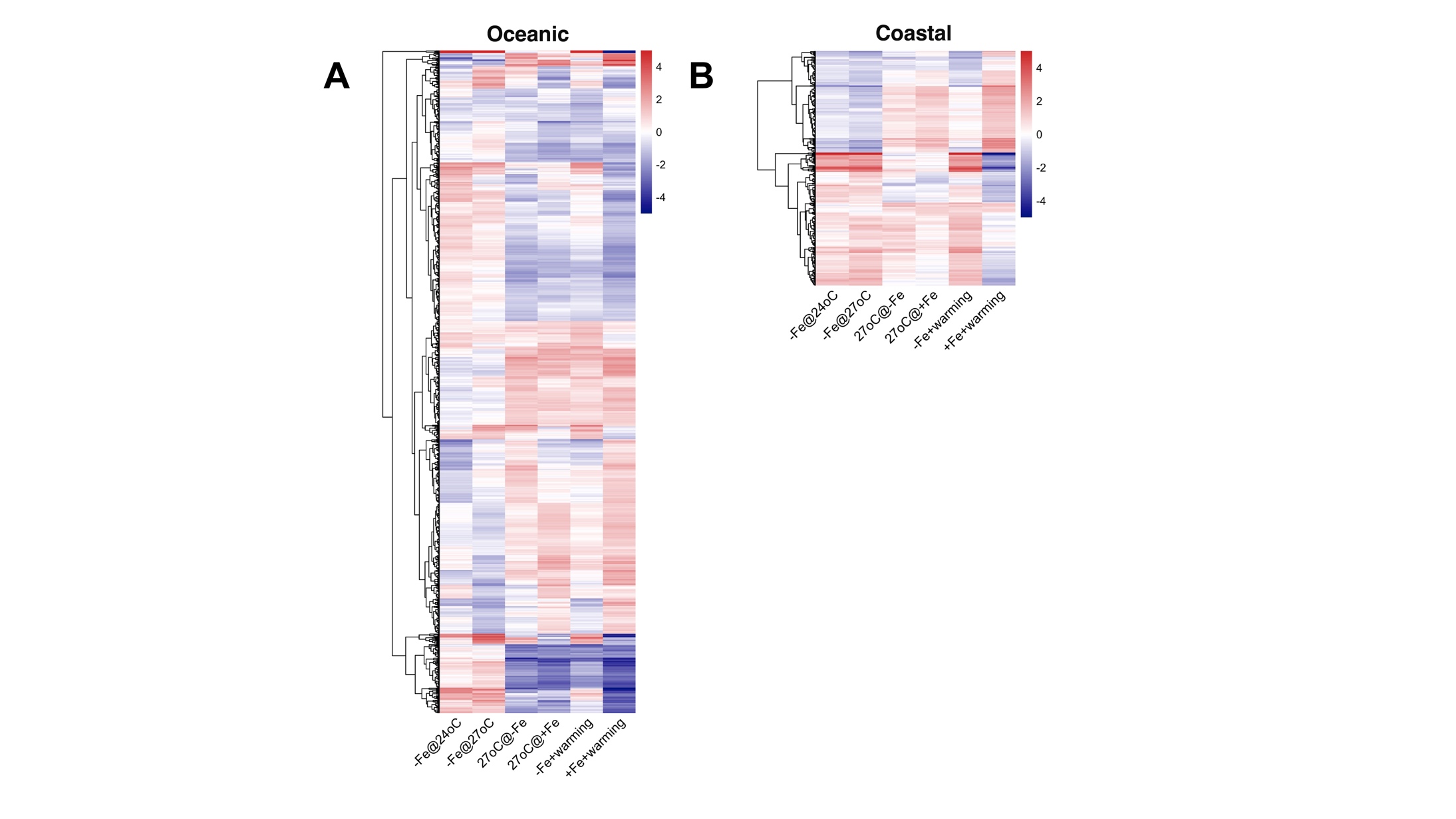
**

**Fig. S2.** The heatmap presents the overall gene expression patterns for all the DEGs from all the comparisons between treatments under different conditions in the oceanic strain (A) and the coastal strain (B). The treatment columns include –Fe@24^o^C, –Fe@27^o^C, 27^o^C@-Fe, 27^o^C@+Fe, -Fe+warming, +Fe+warming. The detailed description of each treatment is indicated in the Method. Each row denotes one gene with ID and annotation listed. The asterisk denotes differential expression with a fold change greater than 2 and an adjusted p-value below the threshold of 0.05. Upregulated genes are represented in red, while downregulated genes are depicted in blue.

**
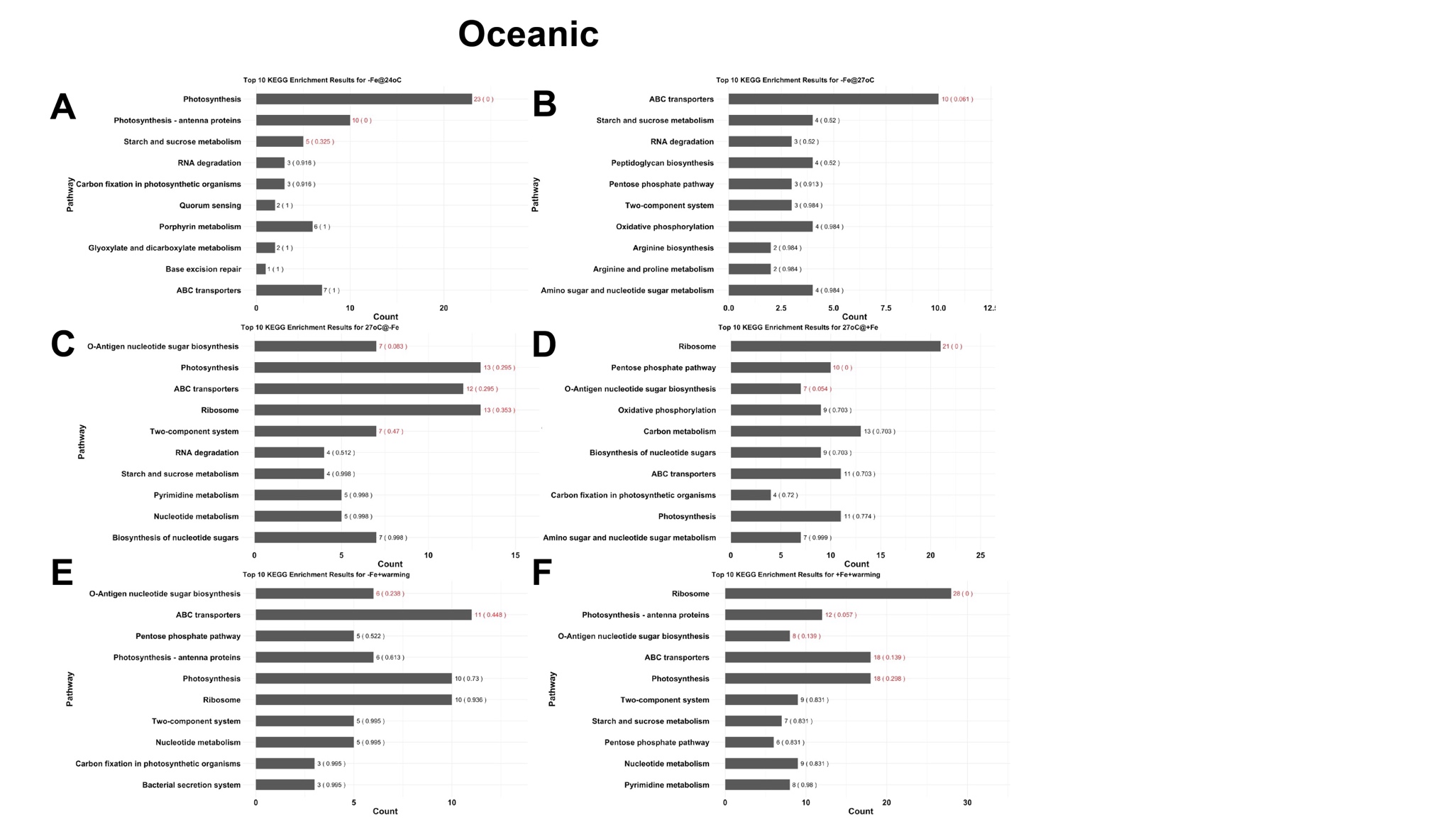
**

**Fig. S3.** The KEGG enrichment analysis was conducted for the oceanic strain. Each figure presents the top 10 most enriched pathways enriched under different conditions ordered by p-value, including –Fe@24^o^C, –Fe@27^o^C, 27^o^C@-Fe, 27^o^C@+Fe, -Fe+warming, +Fe+warming. The detailed description of each treatment is indicated in the Method. The enriched pathways with p-value<0.5 are marked with red.

**
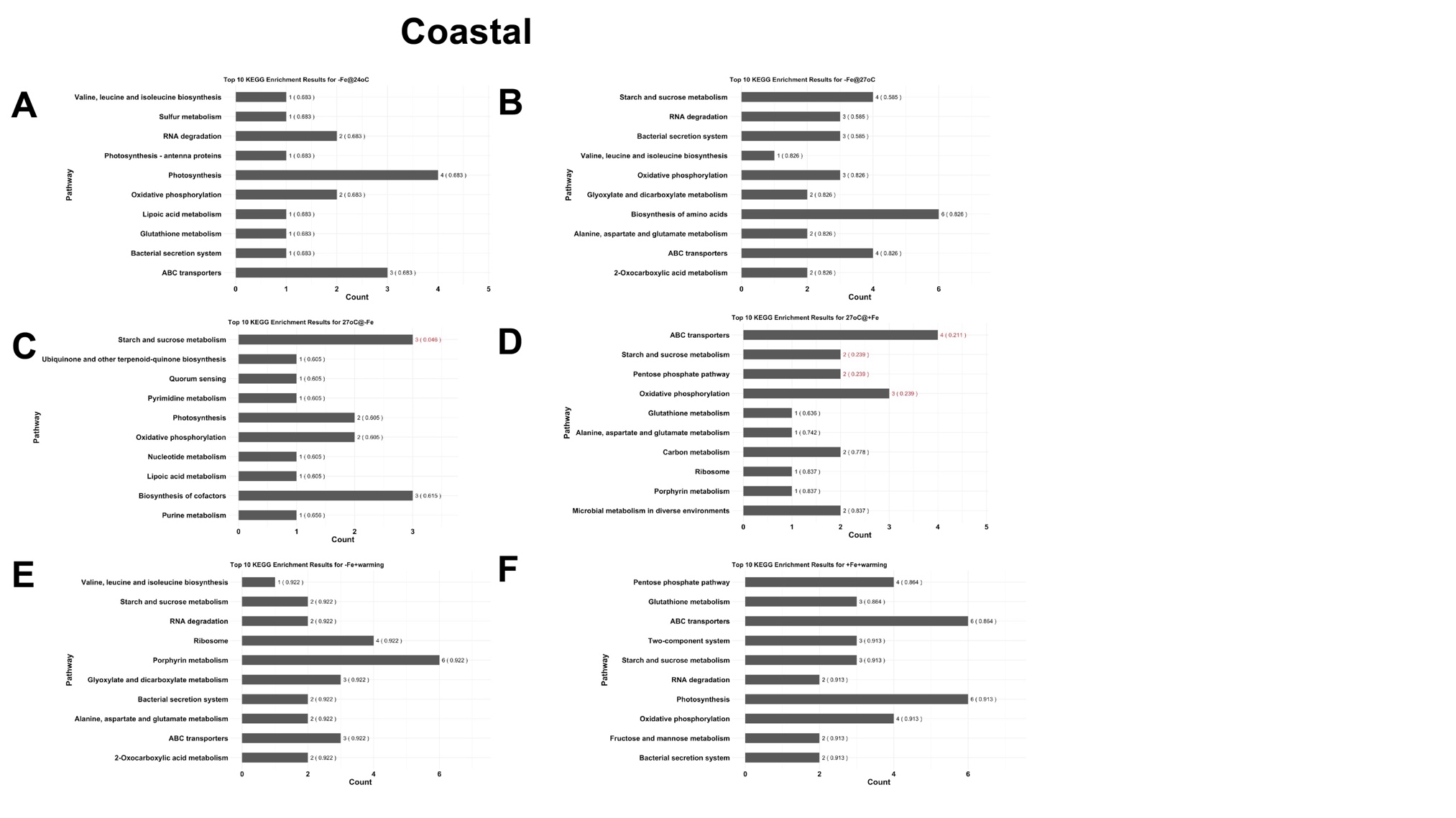
**

**Fig. S4.** The KEGG enrichment analysis was conducted for the coastal strain. Each figure presents the top 10 most enriched pathways enriched under different conditions ordered by p-value, including –Fe@24^o^C, –Fe@27^o^C, 27^o^C@-Fe, 27^o^C@+Fe, -Fe+warming, +Fe+warming. The detailed description of each treatment is indicated in the Method. The enriched pathways with p-value<0.5 are marked with red.

**
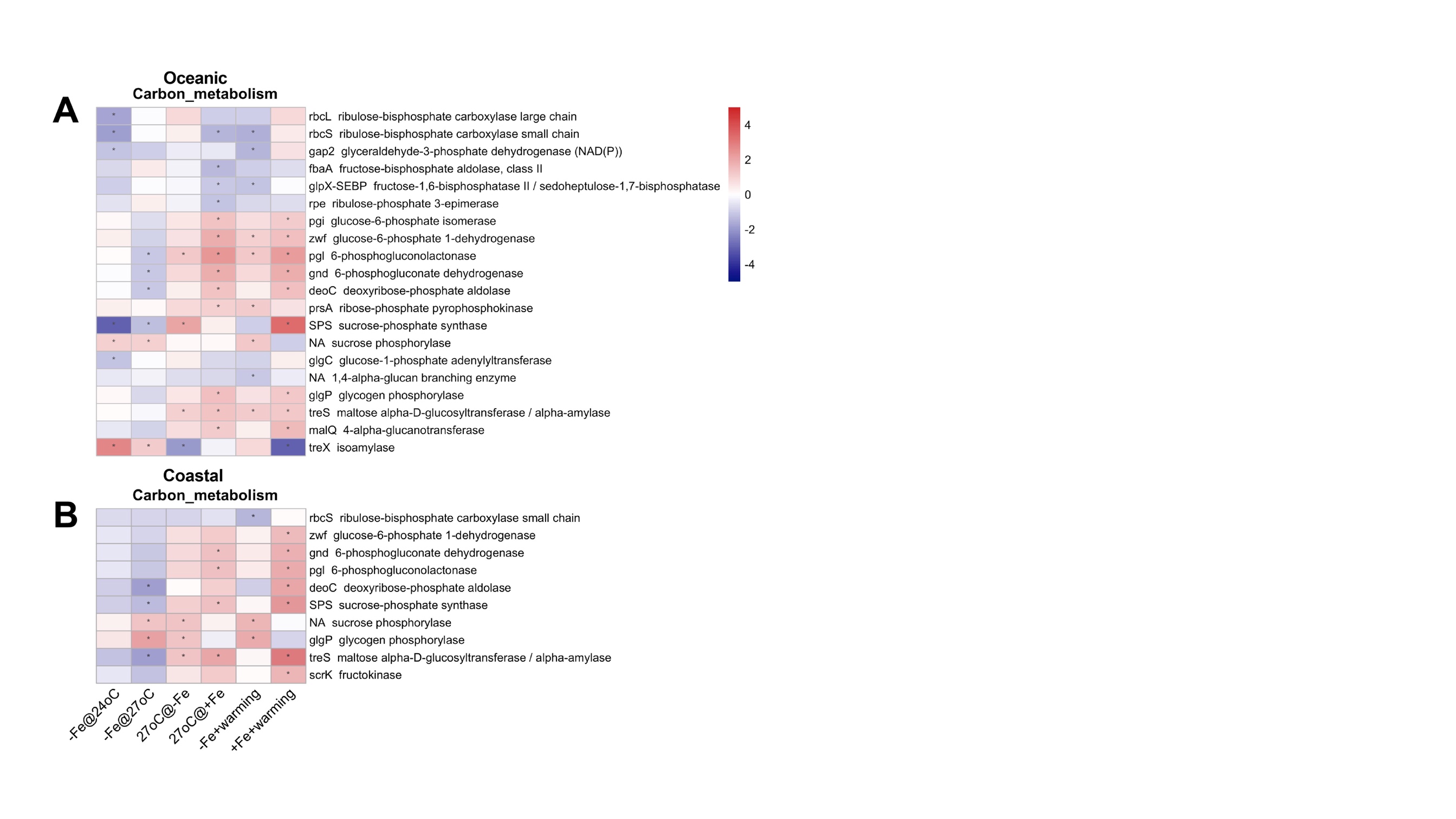
**

**Fig. S5.** The heatmap presents the gene expression patterns involved in carbon metabolism under different treatments in the oceanic strain (A) and the coastal strain (B). The treatment columns include –Fe@24^o^C, –Fe@27^o^C, 27^o^C@-Fe, 27^o^C@+Fe, -Fe+warming, +Fe+warming. The detailed description of each treatment is indicated in the Method. Each row denotes one gene with ID and annotation listed. The asterisk denotes differential expression with a fold change greater than 2 and an adjusted p-value below the threshold of 0.05. Upregulated genes are represented in red, while downregulated genes are depicted in blue.

**
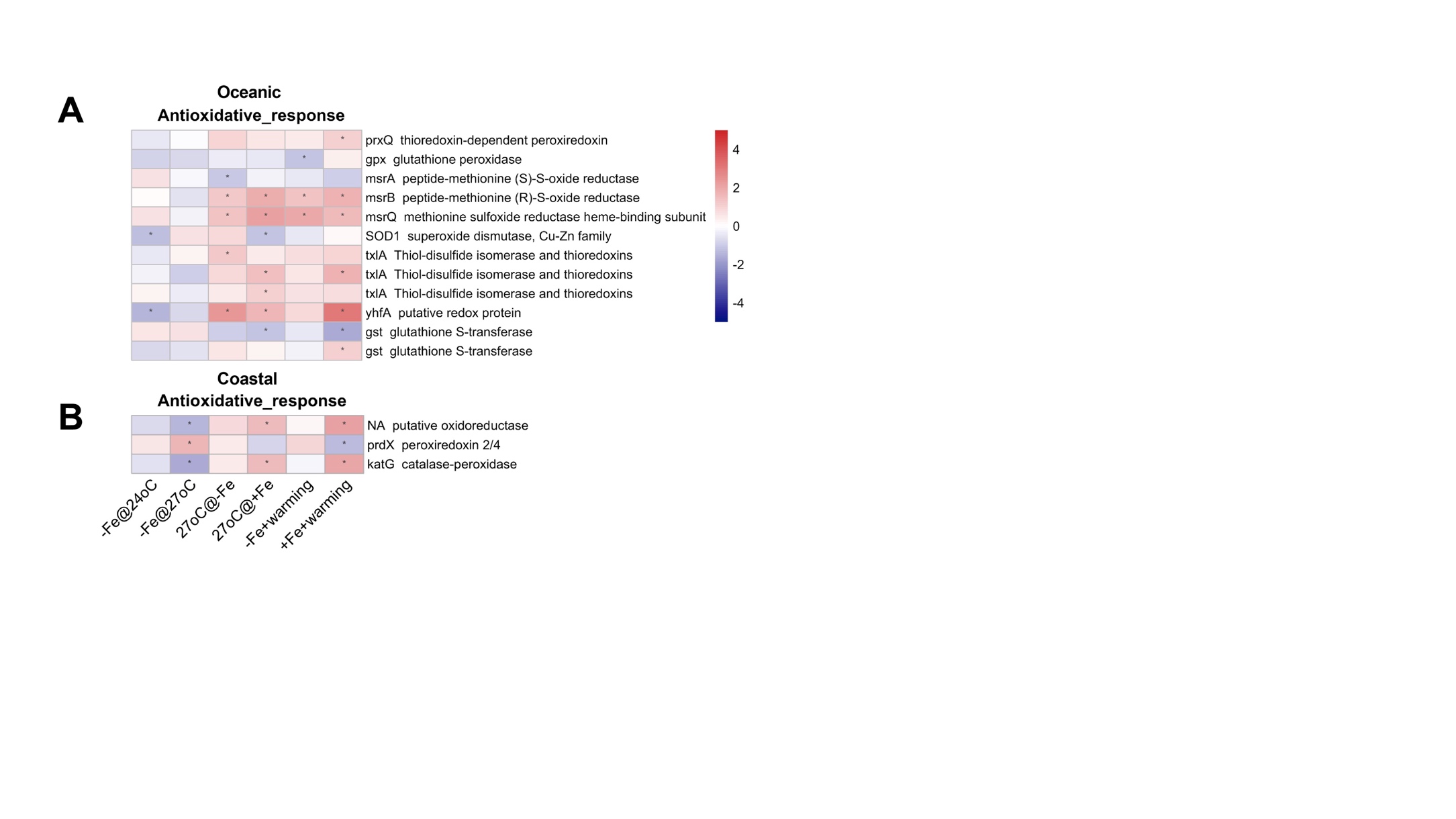
**

**Fig. S6.** The heatmap presents the gene expression patterns involved in antioxidative response under different treatments in the oceanic strain (A) and the coastal strain (B). The treatment columns include –Fe@24^o^C, –Fe@27^o^C, 27^o^C@-Fe, 27^o^C@+Fe, -Fe+warming, +Fe+warming. The detailed description of each treatment is indicated in the Method. Each row denotes one gene with ID and annotation listed. The asterisk denotes differential expression with a fold change greater than 2 and an adjusted p-value below the threshold of 0.05. Upregulated genes are represented in red, while downregulated genes are depicted in blue.

**
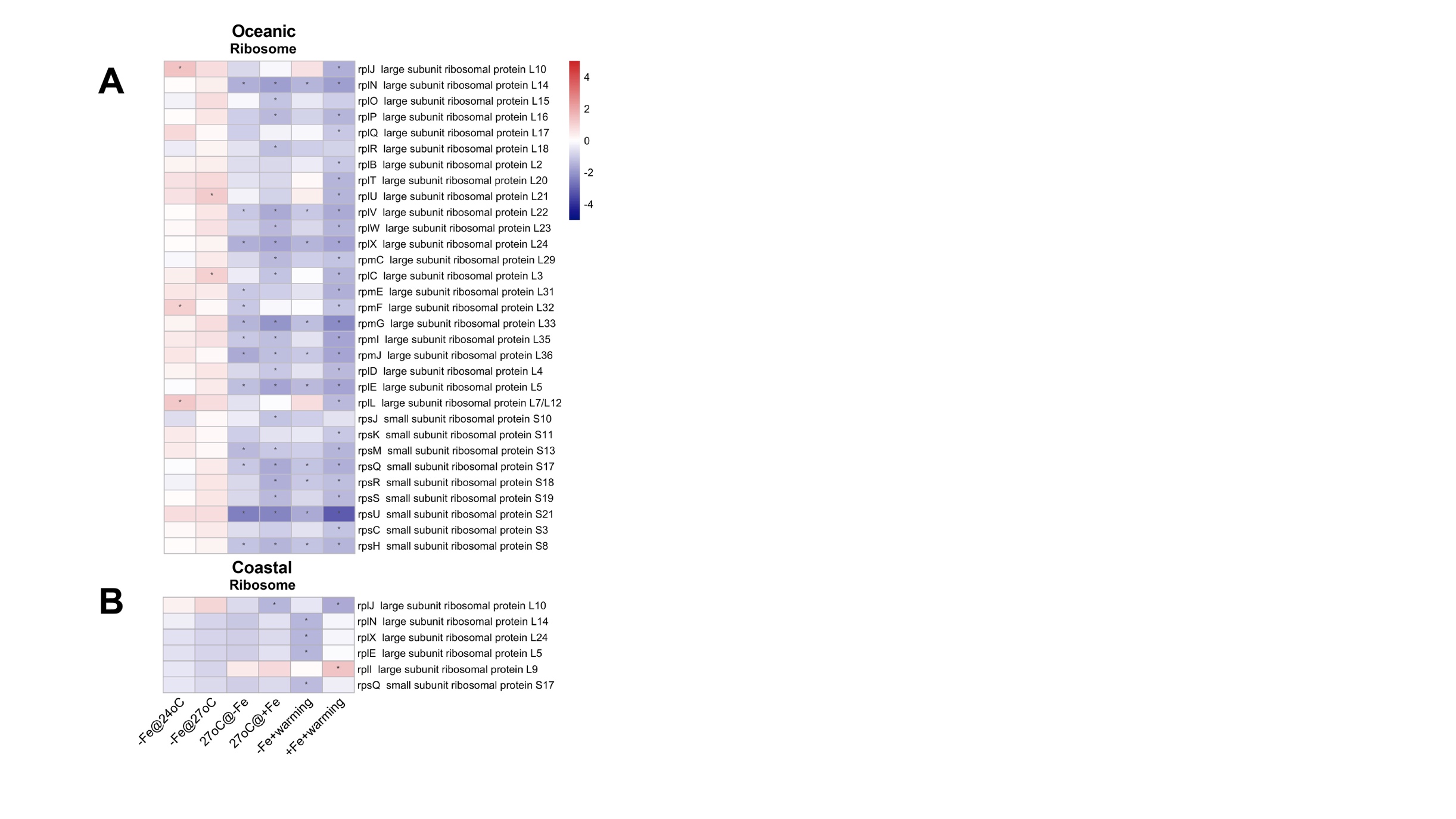
**

**Fig. S7.** The heatmap presents the gene expression patterns involved in ribosome under different treatments in the oceanic strain. The treatment columns include –Fe@24^o^C, –Fe@27^o^C, 27^o^C@-Fe, 27^o^C@+Fe, -Fe+warming, +Fe+warming. The detailed description of each treatment is indicated in the Method. Each row denotes one gene with ID and annotation listed. The asterisk denotes differential expression with a fold change greater than 2 and an adjusted p-value below the threshold of 0.05. Upregulated genes are represented in red, while downregulated genes are depicted in blue.

| Comparison Name | Condition A | Condition B | Description |
| --- | --- | --- | --- |
| 1) -Fe@24°C | 24°C, Fe-limited | 24°C, Fe-replete | Evaluates the effect of Fe limitation at lower temperature (24°C) by comparing Fe-limited and Fe-replete cultures under the same temperature. |
| 2) -Fe@27°C | 27°C, Fe-limited | 27°C, Fe-replete | Evaluates the effect of Fe limitation at high temperature (27°C) by comparing Fe-limited and Fe-replete cultures under the same temperature. |
| 3) 27°C@-Fe | 27°C, Fe-limited | 24°C, Fe-limited | Focuses on the effect of warming when Fe is limiting by comparing high temperature (27°C) to low temperature (24°C), both with Fe limitation. |
| 4) 27°C@+Fe | 27°C, Fe-replete | 24°C, Fe-replete | Focuses on the effect of warming when Fe is replete by comparing high temperature (27°C) to low temperature (24°C), both with Fe repletion. |
| 5) -Fe + warming | 27°C, Fe-limited | 24°C, Fe-replete | Tests the interactive effect of Fe limitation and warming by comparing Fe-limited/high temperature to Fe-replete/low temperature. |
| 6) +Fe + warming | 27°C, Fe-replete | 24°C, Fe-limited | Tests the interactive effect of warming and Fe repletion by comparing Fe-replete/high temperature to Fe-limited/low temperature. |

**Table S1.** A summary of the six treatment comparisons inherent in the experimental design. Each comparison includes its name (as used in the text), the two conditions being compared (including temperature and Fe status), and a brief description of what the comparison represents.

**Table S2 (separate file).** The test details by 2way ANOVA of all the physiology, including particulate organic carbon (POC), particulate organic carbon (PON), Fe quota indicated by Fe to P and Fe to C, and C fixation.

**Table S3 (separate file).** The top 20 upregulated and downregulated DEGs in each comparison between treatments in the oceanic strain.

**Table S4 (separate file).** The top 20 upregulated and downregulated DEGs in each comparison between treatments in the coastal strain.
